# The epidemiology and clinical characteristics of myeloproliferative neoplasms in Malaysia

**DOI:** 10.1186/s40164-018-0124-7

**Published:** 2018-12-17

**Authors:** Yee Yee Yap, Kian Boon Law, Jameela Sathar, Ngee Siang Lau, Ai Sim Goh, Teng Keat Chew, Soo Min Lim, Padmini Menon, Yong Khee Guan, Azlan Bin Husin, Lily Lee Lee Wong, Lee Ping Chew, Sinari Salleh, Kim Yen Goh, Kin Wah Leong, Sen Mui Tan, Tee Chuan Ong, Su Hong Lim, See Guan Toh, Xavier Sim Yoon Han, Syed Carlo Edmund, Jenq Tzong Tan, Kian Meng Chang

**Affiliations:** 1 Department of Haematology, Ampang Hospital, Jalan Mewah Utara, Pandan Mewah, 68000 Ampang, Selangor Malaysia; 2 Clinical Trial Unit, Clinical Research Centre, Ministry of Health, Ampang, Selangor Malaysia; 3 Department of Haematology, Penang General Hospital, George Town, Penang Malaysia; 4 Department of Haematology, Hospital Sultanah Aminah, Johor Bahru, Johor Malaysia; 5 Department of Haematology, Ipoh Hospital, Ipoh, Perak Malaysia; 6 Department of Haematology, Melaka Hospital, Melaka City, Melaka Malaysia; 7 Department of Haematology, Hospital Universiti Sains Malaysia, Kota Bharu, Kelantan Malaysia; 8 Department of Haematology, Queen Elizabeth Hospital, Kota Kinabalu, Sabah Malaysia; 9 Department of Haematology, Sarawak General Hospital, Kuching, Sarawak Malaysia; 10 Department of Haematology, Hospital Raja Perempuan Zainab II Kota Bahru, Kota Bahru, Kelantan Malaysia; 11 Department of Haematology, Ampang Putri Hospital, Ampang, Selangor Malaysia; 12 Department of Haematology, Gleneagles Penang Hospital, George Town, Penang Malaysia; 13 Department of Haematology, Taiping Hospital, Taiping, Perak Malaysia; 14 Department of Haematology, Sunway Medical Centre, Kuala Lumpur, Malaysia

## Abstract

**Background:**

The evolution of molecular studies in myeloproliferative neoplasms (MPN) has enlightened us the understanding of this complex disease consisting of polycythaemia vera (PV), essential thrombocythemia (ET) and primary myelofibrosis (PMF). The epidemiology is well described in the western world but not in Asian countries like Malaysia.

**Materials and methods:**

This retrospective national registry of MPN was conducted from year 2009 to 2015 in Malaysia.

**Results:**

A total of 1010 patients were registered over a period of 5 years. The mean age was 54 years with male predominance. The ethnic distribution revealed that Chinese had a relatively high weighted incidence proportion (43.2%), followed by Indian (23.8%), Malay (15.8%) and other ethnic groups (17.2%). The types of MPN reported were 40.4% of ET (n = 408), 38.1% of PV (n = 385), 9.2% of PMF (n = 93), 3.1% of hypereosinophilic syndrome (HES) (n = 31) and 7.9% of unclassifiable MPN (MPN-U) (n = 80). Splenomegaly was only palpable clinically in 32.2% of patients. The positive JAK2 V617F mutation was present in 644 patients with 46.6% in PV, 36.0% in ET, 9.0% in PMF, and 7.4% in MPN-U, and had significantly lower haemoglobin (p < 0.001), haematocrit (p < 0.001) and white blood cells (WBC) (p < 0.001) than those with negative mutation. Significant differences in platelet and WBC count were detected in ethnic groups and MPN sub-types. There were more arterial thrombosis events seen in those with JAK2 V617F mutation as compared to venous thrombosis events (23.1% vs 4.4%). The bleeding rate was only 6.6%. Among the risk factors, previous thrombosis, old age (≥ 60 years) and hypertension were significantly correlated to positive JAK2 V617F mutation. The arterial thrombosis event is associated with higher presenting HB, HCT and PLT while the bleeding event is associated with lower presenting HB, HCT but higher PLT. The presence of JAK2 V617F mutation is associated with higher risk of arterial thrombosis.

**Conclusion:**

Chinese ethnicity is associated with higher rates of MPN. The history of thrombosis, age ≥ 60 years and hypertension are risk factors that can be correlated to JAK2 V617F mutation. This study is instrumental for policy makers to ensure preventive strategies can be implemented in future.

## Introduction

Myeloproliferative neoplasm (MPN) was distinguished by Polycythaemia Vera Study Group and World Health Organization (WHO) classification as philadelphia (Ph1) chromosome negative essential thrombocythaemia (ET), polycythaemia vera (PV) and primary myelofibrosis (PMF) from Ph1 chromosome positive chronic myeloid leukaemia (CML) [1]. This group of myeloproliferative disorders is characterized by increased proliferation of erythroid, megakaryocytic, or granulocytic cells, often can be complicated by thromboembolic events, and transformation to acute leukemia. Therefore, major causes of death and complications include thrombosis, bleeding, and transformation into overt myelofibrosis or acute myeloid leukemia (AML) [2, 3]. This is a well-known fact nowadays as we know Ph1 chromosome or BCR-ABL 1 gene is the disease specific marker for CML patients. The advent of tyrosine-kinase inhibitor (TKI) has revolutionized this disease.

The discovery of an important somatic mutation in MPN, an activating mutation in the Janus kinase 2 domain (JAK2 V617F) of the erythropoietin (EPO) receptor contributed to the understanding of the pathophysiology, pathogenesis and molecular biology of MPN. The proportion of JAK2 V617F mutation is approximately 95% in PV, 50% to 60% in both ET and PMF [3]. However, MPN is still very poorly understood despite the discovery of JAK2 V617F, calreticulin (CALR), the thrombopoietin receptor (MPL) and many more other gene mutations [4].

The mutational landscape of MPNs has been very impressive lately rendering a substantial insight of the pathogenesis. A study conducted on 103 MPN patients in Korea revealed that JAK2 V617F mutation was correlated with older age, higher neutrophil count, greater rates of organomegaly, thrombotic events and myelofibrosis in ET patients [5].

The development of JAK2 V617F inhibitor, ruxolitinib as the treatment for those with JAK2 V617F myelofibrosis has not been as successful as those CML with TKI. Each type of this complex disease is able to evolve into another type, which makes the diagnosis, risk assessment and therapeutic choices difficult over decades [4]. The morbidity outcome of the disease like arterial thrombosis (AT), venous thrombosis (VT), bleeding as well as the mortality outcome like transformation into aggressive form including bone marrow (BM) failure or acute leukemia are largely unknown.

In terms of annual incidence rate, it was reported as 0.84, 1.03 and 0.47 per 100,000 for PV, ET and PMF respectively based on meta-analysis of 34 studies which were highly heterogenous [6]. Hence, this MPN registry aims to provide more in-depth estimates for regional and global comparison. This study will also enlighten clinicians about the correlations between the allele burden of the JAK2 V617F mutation to the clinical presentations and hematologic findings in MPNs.

## Materials and methods

This MPN registry was conducted from 2009 to 2015, and contributed by 11 participating institutions, namely Ampang Hospital, Selangor (328 cases), Hospital Raja Permaisuri Bainun, Perak (127 cases), Penang General Hospital, Penang (119 cases), Hospital Sultanah Aminah Johor Bahru, Johor (95 cases), Hospital Universiti Sains Malaysia, Kelantan (77 cases), Queen Elizabeth Hospital, Sabah (74 cases), Sarawak General Hospital, Sarawak (58 cases), Gleneagles Hospital, Penang (58 cases), Melaka General Hospital, Melaka (46 cases), Ampang Putri Hospital, Selangor (21 cases), and Hospital Raja Perempuan Zainab II, Kelantan (7 cases). The MPN registry accepted both old and new cases with confirmed diagnosis, therefore the year of diagnosis for MPNs ranged from 1980 to 2015, with approximately 70% of MPNs diagnosed between year 2010 and 2014.

A standard MPN registry form was used to report MPN case, which consisted of information about patient demographics, date of diagnosis, clinical history, sub-types, molecular findings: BM fibrosis, JAK2 V617F mutation, presenting blood parameters: hemoglobin (HB), hematocrit (HCT), platelet (PLT), and white blood cell (WBC), presence of splenomegaly, AT, VT, bleeding, and vasomotor symptoms, and presence of risk factors at presentation: previous AT and VT, old age (≥ 60 years), sex (male), hypertension, dyslipidemia, diabetes mellitus, obesity [body mass index (BMI) > 29.9 kg/m^2^] and smoking status.

All MPN cases were reported by first attending hematologists. The registry forms were all sent to MaxStation Malaysia, a subsidiary entity of The MAX Foundation for data query, update and management. All reported data were reviewed for completeness before being pooled in a final database.

The analysis or report of MPN data were divided into two parts. The first part of analysis mainly involves summarizing the data, descriptive analysis and comparisons based on demographic differences, JAK2 V617F mutation and sub-types. The proportion of MPN cases was adjusted based on Malaysian population composition in year 2016 [7]. Statistical methods applied for analysis of difference include independent t-tests and analysis of variances (ANOVA) for continuous numerical variables, Z-test, Chi square test or Fisher’s exact test for comparing categorical variables or proportions between groups. A result is considered statistically significant if the computed p-value (p) is less than 0.05, for two-sided hypothesis testing with 5% significance level. The second part of analysis was mainly for the survival pattern and clinical outcomes of the cohort.

According to the WHO criteria for PV, the major criteria are HB > 16.5 g/dL in men and > 16 g/dL in women or HCT > 49% in men and > 48% in women or increased red cell mass (RCM); BM biopsy shows hypercellularity for age with trilineage growth, panmyelosis including prominent erythroid, granulocytic and megakaryocytic proliferation with pleomorphic, mature megakaryocytes; presence of JAK2 V617F or JAK2 exon 12 mutation [8]. The minor criteria for PV is subnormal serum EPO level. The diagnosis of PV is made based either all the three major criteria or the first two major criteria and the minor criteria.

On the other hand, the WHO criteria for ET refers to major criteria of PLT count ≥ 450 × 10^9^/L, BM biopsy shows proliferation, mainly of the megakaryocyte lineage, mature megakaryocytes with hyperlobulated nuclei (no significant increase or left shift in neutrophils granulopoiesis or erythropoiesis and increase in reticulin fibers), not meeting WHO criteria for BCR-ABL1 + CML, PV, PMF, myelodysplastic syndromes (MDS) or other myeloid neoplasms, and presence of JAK2 V617F, CALR or MPL mutation. The minor criteria for ET is presence of a clonal marker or absence of evidence of reactive thrombocytosis. Four major criteria or first three major criteria and the minor criterion need to be fulfilled for diagnosis of ET [8].

Similarly, the WHO definition for overt PMF must meet the three major criteria and at least one minor criteria, namely presence of megakaryocytic proliferation and atypia (accompanied by either reticulin or collagen fibrosis grades 2 or 3), not meeting WHO criteria for ET, PV, BCR-ABL1 + CML, MDS or other myeloid neoplasms and presence of JAK2 V617F, CALR or MPL mutation. The minor criteria are anemia not due to comorbid condition, leukocytosis ≥ 11 × 10^9^/L, palpable splenomegaly, increased lactate dehydrogenase (LDH) and leucoerythroblastosis [8].

## Results

**Fig. 1:**
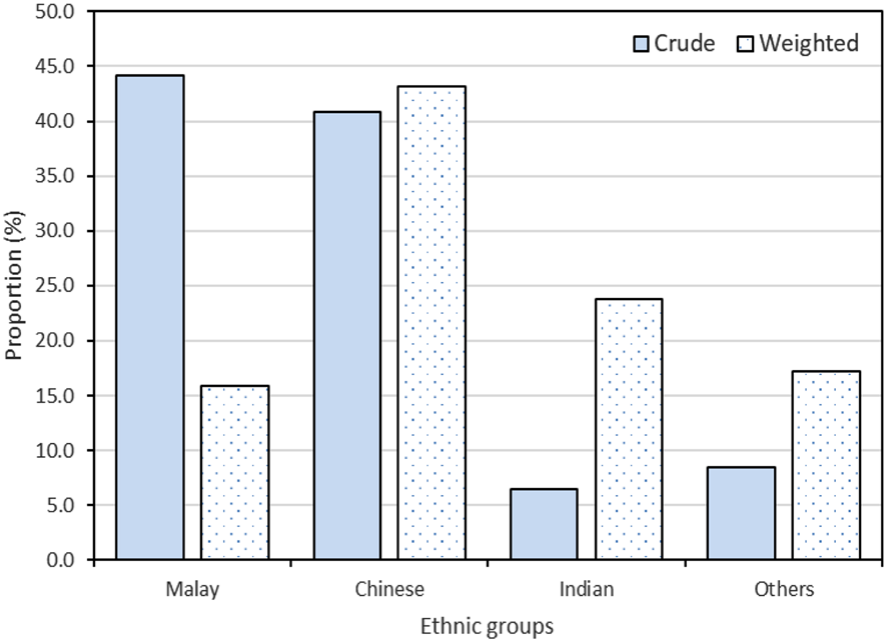
Crude and weighted proportion of MPNs according to ethnic groups in Malaysia. Weighted proportion was calculated based on Malaysia population ethic composition in year 2016

Table 1 summarizes the characteristics of a total of 1010 MPN patients, and according to JAK2 V617F mutation reported by all institutions. The overall MPN cohort presented a mean age of 54.2 years with standard deviation (sd) of 14.88 years and 95% confidence interval (CI) for population mean age of 53.32, 55.16 years. Male MPNs were 3.4% more than female MPNs. Majority of MPN cases were Malay ethnic (44.2%), followed by Chinese (40.8%), Indian (6.4%), native Sabah (4.2%), native Sarawak (1.9%), and other ethnic groups including foreigners (2.5%). However, by considering the weightage of ethnic composition of Malaysia for year 2016, with approximately 61.8% of Malay, 21.2% of Chinese, 6.4% of Indian, and 10.6% of other ethnic populations including foreigners [7], a relatively high adjusted incidence proportion of 43.2% was observed among Chinese population, followed by 23.8% in Indian population, 17.2% in other ethnic groups and only 15.8% in Malay population (Fig. 1).

**Table 1:** The characteristics of MPNs in Malaysia

No	Characteristics	Total MPNs	JAK2 V617F positive^a^	JAK2 V617F negative^a^	p-value
		N (%)	n (%)	n (%)	
1.	Total patients, n	1010	644	222	
2.	Age at diagnosis, year				
	Mean (sd)	54.2 (14.88)	55.9 (13.39)	49.1 (16.59)	< 0.001
	95% CI for population mean	53.32, 55.16	54.90, 56.97	46.87, 51.26	
3.	Sex				
	Female	488 (48.3)	317 (49.2)	99 (44.6)	0.234
	Male	522 (51.7)	327 (50.8)	123 (55.4)	
4.	Ethnic groups^b^				
	Malay	447 (44.2)	310 (48.1)	84 (37.8)	< 0.001
	Chinese	412 (40.8)	253 (39.3)	104 (46.8)	
	Indian	65 (6.4)	32 (5.0)	24 (10.8)	
	Native—Sabah	42 (4.2)	22 (3.4)	5 (2.3)	
	Native—Sarawak	19 (1.9)	13 (2.0)	0 (0)	
	Others	25 (2.5)	14 (2.2)	5 (2.3)	
5.	MPN sub-types				
	ET	408 (40.4)	232 (36.0)	123 (55.4)	< 0.001
	PV	385 (38.1)	300 (46.6)	49 (22.1)	
	PMF	93 (9.2)	58 (9.0)	12 (5.4)	
	HES	31 (3.1)	0 (0)	22 (9.9)	
	MPN-U	80 (7.9)	48 (7.4)	11 (5.0)	
	Others	5 (0.5)	1 (0.2)	2 (0.9)	
	Not documented/missing	8 (0.8)	5 (0.8)	3 (1.3)	
6.	Splenomegaly				
	Yes	325 (32.2)	237 (36.8)	35 (15.8)	< 0.001
	No	680 (67.3)	405 (62.9)	186 (83.8)	
	Not documented/missing	5 (0.5)	2 (0.3)	1 (0.4)	
7.	Presenting vasomotor symptoms^c^				
	Yes	218 (21.6)	154 (23.9)	36 (16.2)	0.015
	No	780 (77.2)	482 (74.8)	185 (83.3)	
	Not documented/missing	12 (1.2)	8 (1.3)	1 (0.5)	
8.	Presenting arterial thrombosis^d^				
	Yes	209 (20.7)	149 (23.1)	28 (12.6)	< 0.001
	No	792 (78.4)	492 (76.4)	193 (86.9)	
	Not documented/missing	9 (0.9)	3 (0.5)	1 (0.5)	
9.	Presenting venous thrombosis^e^				
	Yes	35 (3.5)	28 (4.4)	4 (1.8)	0.082
	No	967 (95.7)	612 (95.0)	217 (97.7)	
	Not documented/missing	8 (0.8)	4 (0.6)	1 (0.5)	
10.	Presenting bleeding^f^				
	Yes	67 (6.6)	40 (6.2)	14 (6.3)	0.956
	No	936 (92.7)	602 (93.5)	207 (93.2)	
	Not documented/missing	7 (0.7)	2 (0.3)	1 (0.5)	
11.	Incidental finding				
	During medical attention for unrelated condition	494 (48.9)	324 (50.3)	109 (49.1)	0.324
	Health screening	118 (11.7)	69 (10.7)	32 (14.4)	
	No	379 (37.5)	242 (37.6)	78 (35.1)	
	Not documented/missing	19 (1.9)	9 (1.4)	3 (1.4)	
12.	BM fibrosis				
	Grade 0	157 (15.5)	94 (14.6)	38 (17.1)	0.202
	Grade 1	86 (8.5)	60 (9.3)	14 (6.3)	
	Grade 2	48 (4.7)	33 (5.1)	12 (5.4)	
	Grade 3	52 (5.1)	40 (6.2)	6 (2.7)	
	Grade 4	21 (2.1)	12 (1.9)	4 (1.8)	
	Suboptimal sample	83 (8.2)	50 (7.7)	24 (10.8)	
	Not done	544 (53.9)	345 (53.6)	121 (54.5)	
	Not documented/missing	19 (2.0)	10 (1.5)	3 (1.4)	

^a^JAK2 V617F mutation result was not reported for 144 patients

^b^Natives from Sabah and Sarawak, and others are grouped together for Chi square test as the expected count must be at least 2

^c^Presenting vasomotor symptoms include headache, transient neurologic or ocular symptoms, distal paraesthesias, erythromelalgia, and others

^d^Presenting arterial thrombosis includes stroke/transient ischemic attack (TIA), acute coronary syndrome, digital gangrene, retinal artery occlusion, mesenteric artery thrombosis, and others

^e^Presenting venous thrombosis includes hepatic or portal vein thrombosis, deep vein thrombosis (DVT), retinal vein thrombosis, sagittal sinus thrombosis, pulmonary embolism (PE), and others

^f^Presenting bleedings include gastro intestinal tract (GIT) unspecified, post-surgical bleed, mucocutaneous, and others

Other characteristics summarized and presented in Table 1 include MPN subtypes: 40.4% of ET, 38.1% of PV, 9.2% PMF, 3.1% of HES and 7.9% of MPN-U; splenomegaly (32.2%), presenting vasomotor symptoms (21.6%), presenting AT (20.7%), presenting VT (3.5%), presenting bleeding (6.6%), and BM fibrosis: grade 0 (15.5%), grade 1 (8.5%), grade 2 (4.7%), grade 3 (5.1%), and grade 4 (2.1%) among MPNs. Due to incompleteness of records or lack of testing, JAK2 V617F mutation results were not reported in about 144 patients, therefore the breakdown of characteristics according to JAK2 V617F mutation was only performed for 866 cases, with 644 positive and 222 negative JAK2 V617F mutation.

Those MPNs with positive JAK2 V617F mutation were associated with higher mean age (55.9 years vs 49.1 years), more female (49.2% vs 44.6%), more Malay ethnic (48.1% vs 37.8%), more PV (46.6% vs 22.1%), more vasomotor symptoms (23.9% vs 16.2%), more AT (23.1% vs 12.6%), more VT (4.4% vs 1.8%) than those MPNs with negative JAK2 V617F mutation (Table 1). CALR and MPL mutation were not tested on those MPNs with no JAK2 V617F mutation.

**Fig. 2:**
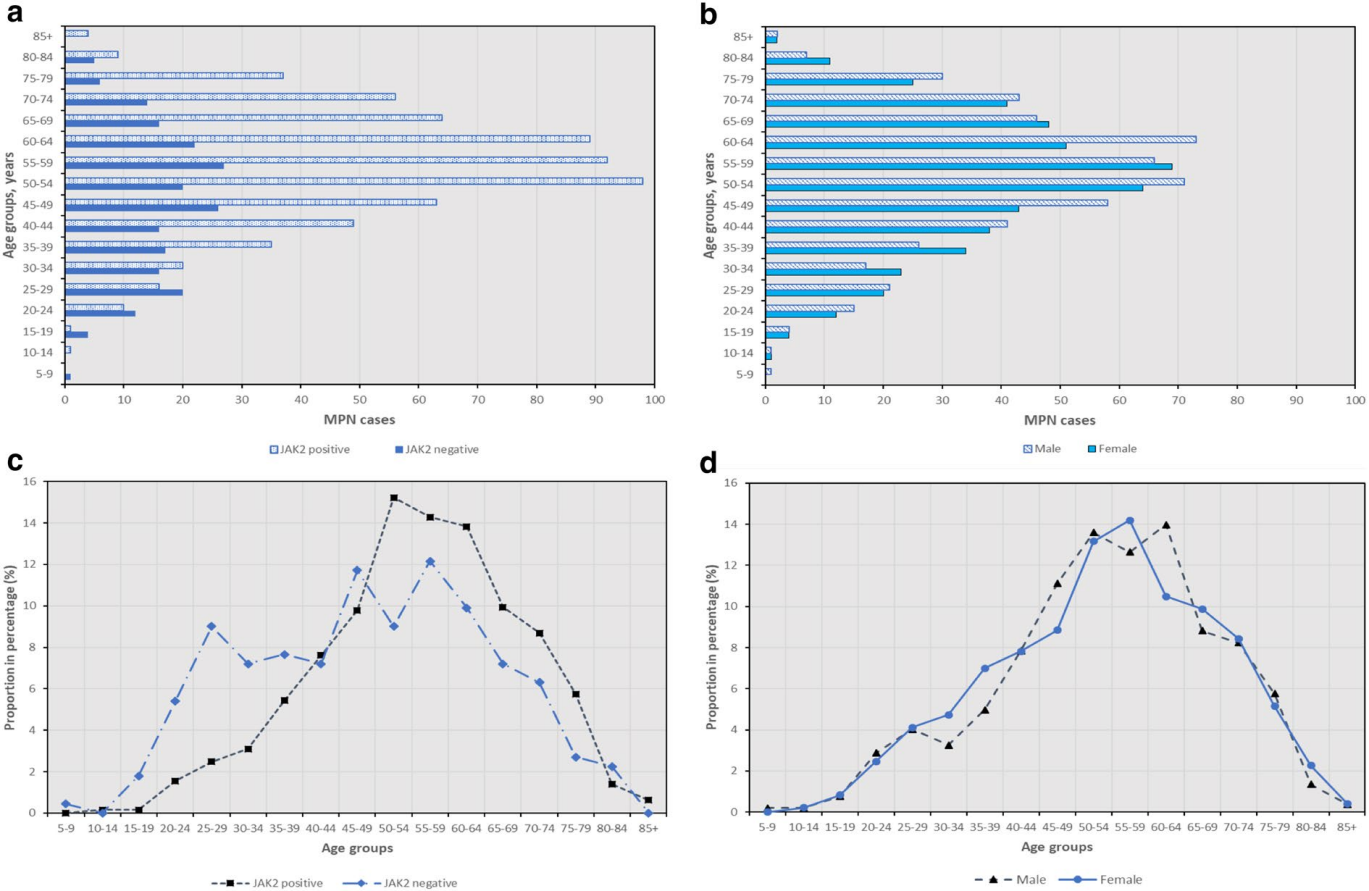
Total MPN cases reported according to **a** JAK2 V617F mutation and **b** sex, and proportion in percentage according to **c** JAK2 V617F mutation and **d** sex

Figure 2 shows the breakdown of total MPNs according to age groups in JAK2 V617F mutation and sex. Our analysis reveals that negative JAK2 V617F mutation presented higher incidence rate from age 45 to 49 years and 55 to 59 years, which was early than those with positive JAK2 V617F mutation (Fig. 2a, c). Patients with positive mutation had higher incidence rate from age 50 to 64 years. Besides, 50 to 64 years in male and age 50 to 59 in female were associated with higher MPN incidence rate than other age groups (Fig. 2b, d), with male having longer age duration (15 years) associated with high incidence rate than female (10 years). However, at the earlier age from 30 to 39 years old, female surpassed male in the MPN (Fig. 2d). CALR and MPL mutation were not tested on those MPNs with no JAK2 V617F mutation.

Table 2 summarizes the analysis of blood counts at presentation for HB, HCT, PLT and WBC according to ethnic groups, JAK2 V617F mutation and sub-types. Statistically significant difference for HB was found among MPNs with JAK2 V617F mutation positive vs negative (mean HB difference = 1.54 g/dL, p < 0.001), and among MPN sub-types (p < 0.001). For sub-types, the mean HB observed were 17.86 g/dL for PV, 13.08 g/dL for ET, 12.29 g/dL for HES, 12.91 g/dL for MPN-U and 9.86 g/dL for PMF, respectively. The mean HB difference between PV and PMF was 8.0 g/dL. A post hoc analysis with Tukey’s test showed that significantly difference for mean HB was found among MPNs for PV when compared with all other sub-types (p < 0.001), and for PMF when compared with all other sub-types (p < 0.001). This implied that patients with PV were associated with significantly higher HB count at presentation as compared to all other MPN sub-types, while the lowest HB count was usually presented in those with PMF. Similar results were obtained for HCT, as both HCT and HB were closely related to each other.

**Table 2:** Analysis of presenting blood counts for MPNs according to sex, race, JAK2 V617F mutation and MPN sub-types

Groups	HB, g/dL		HCT, %		PLT, × 10^9^/L		WBC, × 10^9^/L	
	Mean (sd)	p-value	Mean (sd)	p-value	Mean (sd)	p-value	Mean (sd)	p-value
A. Ethnic groups								
Malay	14.58 (3.99)	0.350	44.96 (13.13)	0.338	716.61 (415.30)	< 0.001	17.75 (13.98)	0.326
Chinese	14.41 (3.59)		44.04 (11.57)		755.20 (444.96)		16.62 (18.09)	
Indian	15.43 (4.06)		46.45 (12.50)		503.92 (305.49)		14.01 (10.64)	
Native—Sabah	14.53 (4.73)		47.57 (14.91)		807.39 (765.28)		19.43 (13.39)	
Native—Sarawak	15.17 (2.62)		46.05 (6.67)		946.26 (515.50)		16.59 (10.18)	
B. JAK2 V617F mutation								
Negative	13.63 (3.43)	< 0.001	41.14 (10.36)	< 0.001	732.84 (498.21)	0.923	12.92 (12.72)	< 0.001
Positive	15.17 (3.80)		46.90 (12.30)		729.58 (404.97)		17.76 (14.13)	
C. Sub-types								
ET	13.08 (2.10)	< 0.001	39.75 (7.24)	< 0.001	1032.20 (424.77)	< 0.001	13.36 (7.74)	< 0.001
PV	17.86 (3.01)		54.73 (10.32)		539.60 (299.68)		16.61 (9.70)	
PMF	9.86 (2.60)		29.88 (8.42)		350.79 (320.11)		22.37 (31.60)	
HES	12.29 (2.71)		36.41 (8.56)		296.63 (241.85)		23.75 (15.24)	
MPN-U	12.91 (3.66)		40.57 (10.95)		741.46 (446.61)		27.68 (23.46)	

Statistically significant difference for PLT was found among different ethnic groups (p < 0.001) and MPN sub-types (p < 0.001). Among the ethnic groups, Indian MPNs presented the lowest mean PLT count (503.92 × 10^9^/L) at presentation, as compared to other ethnic groups. Our post hoc Tukey’s test shows that significant difference for PLT was found among Indian MPNs when compared to Malay (p = 0.003), Chinese (p < 0.001), native Sarawak (p = 0.001), and native Sabah (p = 0.005). For sub-types, ET presented the highest PLT (1032.20 × 10^9^/L), followed by MPN-U (741.46 × 10^9^/L), PV (539.60 × 10^9^/L), PMF (350.79 × 10^9^/L) and HES (296.63 × 10^9^/L). Our post hoc Tukey’s test shows that significant differences exist between all sub-types except for PMF vs HES (p = 0.953).

For WBC, statistically significant difference was found between MPNs with positive vs negative JAK2 V617F mutation (mean WBC difference = 4.84 × 10^9^/L, p < 0.001), and among MPN sub-types (p < 0.001). Among the sub-types, highest presenting WBC was observed in MPN-U (27.68 × 10^9^/L), followed by HES (23.75 × 10^9^/L), PMF (22.37 × 10^9^/L), PV (16.61 × 10^9^/L) and ET (13.36 × 10^9^/L). Our post hoc Tukey’s test shows that significant differences for WBC were between ET when compared to all other sub-types, and between PV when compared to MPN-U (p < 0.001) and PMF (p = 0.005).

Table 3 summarizes the analysis of risk factors profile for total MPNs according to JAK2 V617F mutation. Patients with positive JAK2 V617F mutation were associated with higher odds in presence of previous thrombosis (OR = 2.624, 95% CI 1.717, 4.009), old age (> 60 years) (OR = 1.691, 95% CI 1.215, 2.354) and presence of hypertension (OR = 1.688, 95% CI 1.234, 2.310).

**Table 3:** Analysis of risk factor profile for MPNs according to JAK2 V617F mutation

Risk factors	JAK2 V617F		OR (95% CI)	p-value
	Positive n (%)	Negative n (%)		
Previous thrombosis	183 (28.6)	29 (13.2)	2.624 (1.717, 4.009)	< 0.001
Old age (≥ 60 years)	258 (40.1)	63 (28.1)	1.691 (1.215, 2.354)	0.002
Sex: male	327 (50.8)	123 (55.4)	0.830 (0.611, 1.127)	0.234
Hypertension	327 (50.9)	83 (38.1)	1.688 (1.234, 2.310)	0.001
Dyslipidemia	212 (33.0)	59 (27.2)	1.320 (0.939, 1.855)	0.110
Diabetes	97 (15.1)	39 (17.9)	0.818 (0.545, 1.229)	0.335
Smoking	129 (20.1)	46 (21.1)	0.944 (0.648, 1.376)	0.765
Obese	33 (5.1)	19 (8.8)	0.566 (0.316, 1.011)	0.054

Table 4 showed the analysis of risk factors profile for total MPNs according to MPN subtypes. The risk factor like previous thrombosis and male gender were statistically different in patients with PV followed by MPN-U (p < 0.001). Hypertension, dyslipidaemia became statistically significant risk factors for PV followed by ET (p < 0.001). Smoking was noted to be associated with PV, MPN-U and ET (p = 0.010). PMF was associated significantly with male gender and hypertension with 47.3% and 40.9% respectively whereas only 3.3% of PMF had previous thrombosis. The old age (> 60 years) was observed as statistically significant in association with JAK2 V617F mutation but became non-significant in terms of MPN subtypes (p = 0.139).

**Table 4:** Analysis of risk factor profile for MPNs according to MPN sub-types

Risk factors	MPN sub-types					p-value
	ET n (%)	PV n (%)	PMF n (%)	HS n (%)	MPN-U n (%)	
Previous thrombosis	96 (23.7)	111 (29.1)	3 (3.3)	4 (13.3)	22 (27.5)	< 0.001
Old age (≥ 60 years)	160 (39.2)	132 (34.5)	43 (46.2)	8 (25.8)	31 (39.2)	0.139
Sex: male	166 (40.7)	235 (61.0)	44 (47.3)	24 (77.4)	44 (55.0)	< 0.001
Hypertension	169 (41.7)	223 (58.2)	38 (40.9)	10 (33.3)	29 (36.3)	< 0.001
Dyslipidemia	131 (32.4)	129 (33.7)	15 (16.1)	2 (6.7)	19 (23.8)	< 0.001
Diabetes	66 (16.3)	60 (15.7)	16 (17.2)	5 (16.7)	11 (13.8)	0.976
Smoking	77 (19.0)	91 (23.8)	8 (8.6)	3 (10.3)	18 (22.5)	0.010
Obese	23 (5.7)	28 (7.3)	3 (3.3)	1 (3.3)	5 (6.3)	0.590

**Fig. 3:**
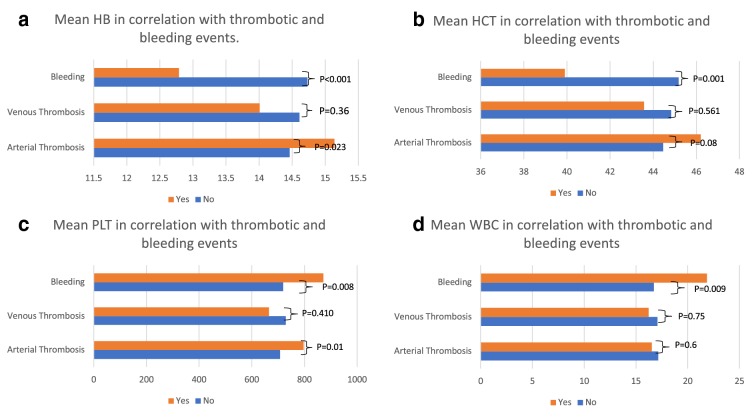
Correlation between full blood count and thrombotic and bleeding events

Figure 3 demonstrated the correlations between full blood counts and thrombotic and bleeding events. In MPN, patients with AT presented higher mean HB that those without AT (mean difference = 0.69, p = 0.023) (Fig. 3a). Similar observation was found for HCT, whereby those with AT presented higher mean HCT (46.2%) when compared to those without AT (Fig. 3b). Both mean HB and mean HCT were significantly lower in the group with bleeding at 12.79 g/dL and 39.90% respectively. Those presented with bleeding events and AT were significantly associated with higher mean PLT (bleeding: p = 0.008; AT: p = 0.01) (Fig. 3c). Similarly, patients with reported bleeding events presented significantly higher mean WBC (p = 0.009).

Table 1 described that JAK2 V617F positivity was present in 149 patients with AT while 28 patients with AT did not have the mutation. The Chi square analysis for the association between JAK2 V617F mutation and AT revealed that MPN patients with positive JAK2 V617F mutation seemed to have 2 times (OR = 2.087, 95% CI 1.352, 3.221) the risk of developing AT as compared to those with negative JAK2 V617F mutation (p = 0.001).

## Discussion

The diagnosis of MPN remains a challenge to all the clinicians nowadays as there are significant overlaps between MDS and MPN of refractory anemia, with ring sideroblasts associated with thrombocytosis. The JAK2 V617F is not specific to MPN alone, in which it can be found in less than 5% of patients with AML, MDS, CML and other myeloid malignancies [9]. The other JAK 2 exon 12 mutation is not reported in this study was described in JAK2 non-mutated PV with predominantly erythroid myelopoiesis [10, 11].

Bone marrow biopsy has become an important major criteria in the WHO 2016 criteria for PV, ET and PMF [8]. In comparison to WHO 2008 criteria and British Committee for Standards in Haematology (BCSH) 2007 criteria for PV, the main emphasis was on HB, HCT and JAK2 V617F mutation [12]. The rationale of integrating BM morphology into the major criteria, for example in PV is to permit a lower level of the HB or HCT threshold for diagnosis. The other main rationale is to improve the difference of masked PV from JAK2-mutated ET and ET from pre-fibrotic early PMF [9]. Nevertheless, there is still lack of standardization of the correct morphological pattern recognition for the MPN subtypes resulting a non-specific classification especially for PV [13].

In the study, most patients with PV were diagnosed with positive JAK2 V617F mutation (46.6%) whereas 22.1% of them were without the mutation, eight cases of MPN had missing information regarding the molecular studies status and possibly diagnosed based on the BM findings hence the BM morphology is a very helpful diagnostic tool despite the diagnostic validity. There is a lot of debate about the application of the HB and HCT levels together with increment of RCM 25% above mean predicted value may lead to under diagnosis of PV whose HB and HCT levels are below the WHO criteria [9]. Hence, the term masked PV (mPV) is used for those with JAK2-mutated patients but with pre-polycythaemic disease manifestation and BM morphology concordant with PV plus raised HB levels between 16.0 and 18.5 g/dL for men and 16.5 g/dL for women. It is important to recognize this group of patients because of higher risk of thrombosis demonstrated in 66 JAK2-mutated patients < 40 years of age compared to a control group of 97 cases with overt PV as there was less frequent use of phlebotomies or cytoreductive treatment for those so called mPV [14]. Barbui et al. [15] pointed out in their study that a worsening overall survival in mPV patients versus those with overt PV according to the WHO (p = 0.01) and the BCSH (p = 0.0019) guidelines.

Essential thrombocythemia has always been incorporated the BM biopsy in the WHO diagnostic criteria and lowering the PLT count of ≥ 600 × 10^9^/L to ≥ 450 × 10^9^/L in the diagnostic guidelines by the WHO and BCSH as the use of the high threshold value is not consistent with the 95th percentile for normal PLT count being below 400 × 10^9^/L [9, 16]. It is important to differentiate the ET from prefibrotic myelofibrosis (prePMF) pertaining to significantly worse survival rates, leukemic transformation rates and rates of progression to overt myelofibrosis in prePMF compared to ET [17, 18]. The prePMF is classified by WHO as major criteria: megakaryocytic proliferation and atypia, without reticulin fibrosis > grade 1, accompanied by increased age-adjusted BMA cellularity together with granulocytic proliferation and decreased erythropoiesis; not meeting the WHO criteria for BCR-ABL1 CML, PV, ET, MDS or other myeloid neoplasms plus presence of JAK2 V617F, CALR or MPL mutations [8]. The minor criterion is either anemia, leucocytosis (WBC ≥ 11 × 10^9^/L), palpable splenomegaly or LDH increased. All the three major criteria and at least one minor criteria are required for prePMF [8]. This condition is not mentioned in our results, but in the BM fibrosis, 243 of patients not having greater than grade 1 BM fibrosis with 24% acquired JAK2 V617F mutation. There might be potential prePMF presented as thrombocytosis and was categorised as ET in our study. This is evident in the mean PLT count for our cohort which is more than 1000 × 10^9^/L as an extreme thrombocytosis probably a marker for occult early prefibrotic MF [18].

In our study, Chinese ethnic showed higher incidence in comparison to Malay, Indian and other ethnic groups. The presence of JAK2 V617F mutation is linked to an inherited JAK2 46/l haplotype which is recognised to be a risk factor for MPN in Caucasian population [19]. Zhang et al. [19] revealed that the JAK 2 46/1 haplotype is also present in Chinese population as a risk factor for MPN and those with GG genotype in rs12340895 locus are frequently associated with JAK2 V617F mutation. Junko et al. demonstrates C allele of JAK2 rs4495487 plus the 46/1 haplotype is associated significantly with the occurrence of JAK2 V617F positive and JAK2 V617F negative MPNs in the Japanese population [20]. There has been good evidence that Japanese is genetically related to people from South-East Asia like China and Korea. The JAK2 46/1 haplotype which is also being referred as “GGCC” is the most commonly known susceptible allele for sporadic MPNs with JAK2 V617F mutation, indicating high probability of inheritance [21]. The Chinese population in Malaysia is mostly originated from Southern China which might explain the highest incidence when compared to other ethnic groups.

Malay ethnic has the lowest prevalence of MPN despite being the largest ethnic population in Malaysia. The early stages of MPN are asymptomatic so many patients may not seek treatment until they become symptomatically unwell. In Malaysia, the primary care system has not been well-developed, unlike western countries in which there will be blood test done on the patients yearly for health check-up. Majority of Malays are residing in rural area resulting a more inconvenient access to better equipped health care facilities. Whereas most of the Chinese are residing close to medical amenity in the urban area hence may result in higher screening rate among them. Nevertheless, this registry had included district hospital with malay being the majority in the area. The observation of Malay having low prevalence of MPN could still be possibly true among all the three main ethnics in Malaysia.

The MPN is associated with increased risk of thrombosis as illustrated in our study. The pathogenesis of the acquired thrombophilia state in ET and PV is abnormalities of MPN-clone-derived blood cells such as erythrocytes, PLTs and leucocytes with prothrombotic features and inflammatory response of normal vascular cells to insult of cytokines plus mediators released by malignant cells resulting in pro-coagulant state [22, 23]. This is characterized by high concentrations of plasma markers of blood clotting like thrombin-antithrombin complex, prothrombin fragment 1 + 2 and D-dimer and vascular endothelium activation like thrombomodulin and von Willebrand factor/factor VIII [22]. The German MPN registry stated that deep vein thrombosis (31.5%) was the most common thromboembolic event followed by cardiac events (27.7%) [24]. It seems like our study reveals there were more AT events than VT. Stephan et al. pointed out that only 30% of venous thromboembolism in MPN patients, which is less common than arterial thromboembolism [25]. The European Collaboration on Low-Dose Aspirin (ECLAP) demonstrates cardiovascular death attributed to 41% of all mortality among the PV patients in which the cause of death were mainly coronary heart disease (15%), congestive heart failure (8%), non-hemorrhagic stroke (8%) and pulmonary embolism (8%) [22, 26]. The cumulative incidence of all thromboembolic events amounts to 2.5–5.0% per patient year in PV whereas 1.9–3.0% per patient year in ET. The prevalence is documented to be ranging 11–39% in PV and 8–29% in ET [25]. This is quite in concordance with our study the previous thrombosis rate was highest in PV followed by MPN-U and ET. The reason for MPN-U had the second highest thrombotic event in our study could be because the possibility of splanchnic vein thrombosis that was not recorded. The German MPN registry shows the most frequent rates of splanchnic vein thrombosis in 60% of MPN-U [24].

Positive JAK2 V617F mutation MPN is associated with longer duration of disease, higher HB level, higher leucocyte count, lower PLT count and higher rate of thrombosis, haemorrhage and fibrosis in comparison to wild-type JAK2 mutation [27]. There is a higher risk of AT in ET (HR 2.57, 95% CI 1.27–5.19) in relation to JAK2 V617F mutation [28]. This is observed in our study. Most of the studies in the west demonstrated a higher rate of venous thrombosis in comparison to arterial thrombosis [29]. The plausible explanation could be inadequate venesection, non-compliance to treatment and low accessibility to ruxolitinib. This observation is instrumental to Asian patient with MPNs which needs to be investigated prospectively.

The PV is related to higher thrombotic risk compared to ET in view of high allele burden which can present in 20–30% of homozygous JAK2 V617F patients [27]. The independent risk factor for AT in MPN is leucocytosis but was not significant in our study (p = 0.6) [22, 30]. There is 70% increase of myocardial infarction in PV patients presented with a WBC count > 15 × 10^9^/L in comparison to those with WBC count < 15 × 10^9^/L [31]. Instead, our study revealed thrombocytosis is associated significantly with AT (p = 0.01). Old age of more than 60 years is significant in our MPN patients with positive JAK2 V617F mutation. The age more than 60 years and previous thrombosis were associated with higher hazard ratio (HR) (1.5 and 1.93, respectively) in developing major thrombosis [22, 32].

The conventional risk factor for atherosclerosis such as hypertension, dyslipidemia and smoking are found to be significantly associated with MPN sub-types. It is believed that the presence of these risk factors can predispose a low-risk ET patient to intermediate or high-risk category for thrombosis [22]. The IPSET-thrombosis study provides a 2-tiered low- and high-risk categories based on the presence of either age > 60 years or history of thrombosis in predicting thrombosis among ET patients [33]. This slowly evolves to a 3-tiered prognostic model based on multivariable-analysis-derived HR to age > 60 years (HR = 1.5; 1 point), thrombosis history (HR = 1.9; 2 points), cardiovascular risk factors (HR = 1.6; 1 point), and JAK2 V617F (HR = 2.0; 2 point) into either low risk < 2 points; intermediate risk = 2 points or high risk > 2 points [33].

Acquired von Willebrand Syndrome (AvWS) has been well described with MPN with extreme thrombocytosis. The risk of major haemorrhage is significantly raised (HR = 3.7) when the PLT counts rise above 450 × 10^9^/L in comparison to those with PLT within normal range [22]. There is approximately tenfolds risk of bleeding with PLT count beyond 1250 × 10^9^/L [34]. The mean PLT in our ET patients is above 1000 × 10^9^/L. This might be the reason for the strong correlation between the PLT count and the haemorrhagic events (p = 0.008), but the rates of bleeding in our cohort is not as common as arterial thrombosis. The pathogenesis of the bleeding is multifactorial, essentially based on the absolute PLT count, whereby there is increasing proteolysis of von Willebrand factor (vWF) by ADAMTS-13 leading to selective loss of large vWF multimers [25]. Other observations include acquired storage pool defect, the increased activation level, and reduced surface density of certain receptors [25, 35].

The main limitation to this study is the retrospective cross-sectional analysis of the data despite the sumptuous number of patients. This includes the incomplete records, recall bias as well as missing important information that results in great difficulty to examine the cause and effect. There was no data on CALR and MPL mutations in this study resulting in incompleteness of the mutational analysis of the MPN cohort. The lack of treatment data also precludes the analysis in the aspect to be done in these patients. Nevertheless, this registry is important in terms of providing a real-world overview of the MPN characteristics including the subtype and complications in a developing country of South East Asia. Most of the MPN registries were conducted in western countries like German SAL-MPN-registry, Swedish Cancer Registry and United States National Cancer Institute’s (NCI) SEER-18 Program on MPNs [2, 24, 36]. The only Asian retrospective study that was available from one centre in Korea and it had only 103 subjects [5]. There has not been such a large-scale registry developed to capture the MPN epidemiology in Asia yet. So, we will be the pioneer to report the eastern clinico-hematologic parameters of MPNs in addition to the plethora of scientific information that is already available in the western literature.

## Conclusion

Chinese ethnicity is significantly associated with higher rate of Ph1-negative MPN in Malaysia. The previous history of thrombosis, advanced age ≥ 60 years and hypertension are strong risk factors correlated with JAK2 V617F detection in our population. Other cardiovascular risk factors including hypertension, dyslipidemia and smoking as well as being a male are significantly correlated with higher rates of PV in this study. The arterial thrombosis event is associated with higher presenting HB, HCT and PLT while the bleeding event is associated with lower HB, HCT but higher PLT at presentation. The presence of JAK2 V617F mutation is associated with higher risk of arterial thrombosis. This study provides us insight into the risk stratification of patient with MPN in relation to JAK2 V617F mutation for the future development of preventive strategies. Future direction should look into the mortality and outcome of MPN in Malaysia.

## Abbreviations

MPN: myeloproliferative neoplasms
PV: polycythaemia vera
ET: essential thrombocythaemia
PMF: primary myelofibrosis
EPO: erythropoietin
JAK 2: Janus kinase 2 domain
MPN-U: myeloproliferative neoplasms unclassifiable
CML: chronic myeloid leukemia
AT: arterial thrombosis
VT: venous thrombosis
WBC: white blood cells
HB: haemoglobin
PLT: platelet
mPV: masked polycythaemia vera
HCT: haematocrit
BM: bone marrow
CALR: calreticulin
vWF: von Willebrand Factor
AvWS: acquired von Willebrand syndrome
ADAMTS-13: a disintegrin-like and metalloprotease with thrombospondin type 1 motif 13
